# Modes of Inhibition of ****α****-Amylase and ****α****-Glucosidase by Aqueous Extract of *Morinda lucida* Benth Leaf

**DOI:** 10.1155/2013/527570

**Published:** 2013-12-24

**Authors:** M. I. Kazeem, J. O. Adamson, I. A. Ogunwande

**Affiliations:** ^1^Department of Biochemistry, Lagos State University, PMB 0001, LASU Post Office, Lagos, Nigeria; ^2^Department of Chemistry, Lagos State University, PMB 0001, LASU Post Office, Lagos, Nigeria

## Abstract

Diabetes mellitus is a metabolic disorder of glucose metabolism. The management of blood glucose level is the hallmark in the treatment of this disease. This may be achieved through the use of oral hypoglycemic drugs such as biguanides, insulin secretagogues, and **α**-glucosidase inhibitors. The purpose of the present study was to investigate the inhibitory effect of *Morinda lucida* leaf extracts on the activities of **α**-amylase and **α**-glucosidase. This was performed using **α**-amylase from *Aspergillus oryzae* and **α**-glucosidase from *Saccharomyces cerevisiae.* Aqueous extract of *Morinda lucida* gave the highest percentage yield (9.99%) of the plant out of the three extracts (compared to acetone and ethanolic extracts) and possesses the highest inhibitory activity against **α**-amylase (IC_50_ value of 2.30 mg/mL) and **α**-glucosidase (IC_50_ value of 2.00 mg/mL). Kinetic analysis revealed that the aqueous extract of this plant leaf inhibited the **α**-amylase competitively but displayed mixed noncompetitive mode of inhibition towards **α**-glucosidase. It can be concluded that aqueous extract of *Morinda lucida* exhibited the best inhibitory activity on the two enzymes studied and the presence of phytochemicals like flavonoids, saponins, and tannins may have contributed greatly to the inhibitory activity of the plant extract.

## 1. Introduction

*Morinda lucida* Benth is a medium-sized tree used as a medicinal plant in West Africa (especially in Nigeria). The leaves are used in the treatment of fever, malaria, and diabetes [1]. Decoctions of the roots, barks and leaves are recognized remedies against different types of fever, including yellow fever and malaria [2]. In some cases, the plant is employed in the treatment of diabetes, hypertension, cerebral congestion, dysentery, stomach ache, ulcers, leprosy, and gonorrheal [3]. Infusion of the stem bark, the root, and leaves serves as a remedy for severe jaundice, malaria, and diabetes [4]. Previous studies had shown the hypoglycemic and antihyperglycemic potentials of *Morinda lucida* Benth extracts [5, 6].

Diabetes mellitus is a complex disease that is characterized by gross derangement in carbohydrate, protein, and fat metabolism. It is a progressive metabolic disorder of glucose metabolism that eventually leads to micro- and macrovascular changes causing secondary complications that are difficult to manage [7]. Type 1 diabetes results from inadequate synthesis of insulin by *β*-cells of the pancreas, while type II diabetes is characterized primarily by insulin resistance (a condition in which peripheral cells do not respond normally to insulin) or *β*-cell dysfunction [8].

Alpha-amylase is a prominent enzyme found in the pancreatic juice and saliva which breaks down large insoluble starch molecules into absorbable molecules [9]. On the other hand, mammalian *α*-glucosidase in the mucosal brush border of the small intestine catalyzes the end step of digestion of starch and disaccharides that are abundant in human diet [10]. Inhibitors of *α*-amylase and *α*-glucosidase delay the breaking down of carbohydrates in the small intestine and diminish the postprandial blood glucose excursion [11].

An effective means of lowering the levels of postprandial hyperglycemia have been offered by *α*-amylase and *α*-glucosidase inhibitors [12]. Several inhibitors of *α*-amylase and *α*-glucosidase has been isolated from medicinal plants to serve as an alternative drug with increased potency and lesser adverse effects than existing synthetic drugs [12, 13]. Though several studies showed the antidiabetic potential of *Morinda lucida* [5, 6] no previous report has been given on the mechanism by which it exerts this effect. We have also published an article on the *α*-glucosidase inhibitory potentials of some Nigerian medicinal plants [14]. As a followup to this, the aim of this study was to evaluate the effect of *Morinda lucida* leaf extracts on the activities of *α*-amylase and *α*-glucosidase as well as determination of modes of inhibition of these enzymes.

## 2. Materials and Methods

### 2.1. Plant Material

The leaf of *Morinda lucida* was obtained from Badagry Area of Lagos in Nigeria in July 2012. It was identified and authenticated by Dr. A. B. Kadiri of the Department of Botany, University of Lagos, Akoka, Lagos, Nigeria, and voucher specimen (LUH 4723) was deposited in the University herbarium.

### 2.2. Chemicals and Reagents

Alpha-amylase from *Aspergillus oryzae*, *α*-glucosidase from *Saccharomyces cerevisiae,* and paranitrophenyl-glucopyranoside were products of Sigma-Adrich Co., St Louis, USA, while starch soluble (extra pure) was obtained from J. T. Baker Inc., Phillipsburg, USA. Other chemicals and reagents were of analytical grade and water used was glass distilled.

### 2.3. Preparation of Plant Extracts

Fresh leaves of *Morinda lucida* were cut and washed with water to remove all contaminants; they were dried under room temperature and grounded to powder. The powdered leaves were divided into three portions and each portion was extracted with acetone, ethanol or water. They were all left to steep in covered containers for 24 hrs; the resulting infusions were decanted, filtered. and evaporated in a rotatory evaporator (Cole Parmer SB 1100, Shangai, China). The extracts were freeze dried using Virtis Bench Top (SP Scientific Series, USA) freeze dryer. Dried extracts were weighed and dissolved in 10% dimethylsulphoxide to yield a stock solution from which lower concentrations were prepared.

### 2.4. Phytochemical Screening

Phytochemical compositions of the leaves were determined using the methods variously described by Trease and Evans [15] and Sofowora [16].

*Test for Anthraquinones.* 5 mL of chloroform was added to 0.5 g of the plant extracts of each specimen. The resulting mixture was shaken for 5 min after which it was filtered. The filtrate was then shaken with equal volume of 10% ammonia solution. The presence of a bright pink colour in the aqueous layer indicated the presence of anthraquinones.

*Test for Flavonoids.* A portion of the plant extract was heated with 10 mL of ethyl acetate over a steam bath for 3 min. The mixture was filtered and 4 mL of the filtrate was shaken with 1 mL of dilute ammonia solution. Development of yellow colouration was an indication of the presence of flavonoids.

*Test for Reducing Sugar.* To about 1 g of each plant extract in the test tube, 10 mL distilled water was added and the mixture boiled for 5 min. The mixture was filtered while hot and the cooled filtrate made alkaline to litmus paper with 20% sodium hydroxide solution. The resulting solution was boiled with an equal volume of Benedict qualitative solution on a water bath. The formation of a brick-red precipitate depicted the presence of reducing compound.

*Test for Saponin.* Approximately 2 g of plant extract was boiled in 20 mL of distilled water in a water bath and filtered. Next, 10 mL of the filtrate was mixed with 5 mL of distilled water and shaken vigorously and observed for a stable persistent froth. The frothing was mixed with 3 drops of olive oil and shaken vigorously again and then observed for the formation of emulsion as an indication of saponin.

*Test for Steroids.* In this test, 2 mL of acetic anhydride was added to 0.5 g of plant extract with 2 mL concentrated H_2_SO_4_. The colour change from violet to blue or green is an indication of steroids.

*Test for Tannins.* In the test for tannins, 0.5 g of plant extract was boiled in 20 mL of water in a test tube and filtered. Few drops of 0.1% ferric chloride was added and observed for a brownish green or blue black colouration as an indication of tannins. 

*Test for Terpenoids.* In brief, 0.5 g of plant extract was mixed with 2 mL chloroform and 3 mL H_2_SO_4_ was carefully added to form a layer. A reddish brown colouration of the interface was an indication of terpenoids.

### 2.5. Alpha-Amylase Inhibitory Assay

This assay was carried out using a modified procedure of McCue and Shetty [17]. A total of 250 *μ*L of extract (1.25–10 mg/mL) was placed in a tube and 250 *μ*L of 0.02 M sodium phosphate buffer (pH 6.9) containing *α*-amylase solution (0.5 mg/mL) was added. This solution was preincubated at 25°C for 10 min, after which 250 *μ*L of 1% starch solution in 0.02 M sodium phosphate buffer (pH 6.9) was added at timed intervals and then further incubated at 25°C for 10 min. The reaction was terminated by adding 500 *μ*L of dinitrosalicylic acid (DNS) reagent. The tubes were then incubated in boiling water for 5 min and cooled to room temperature. The reaction mixture was diluted with 5 mL distilled water and the absorbance was measured at 540 nm using spectrophotometer. A control was prepared using the same procedure replacing the extract with distilled water. The *α*-amylase inhibitory activity was calculated as percentage inhibition:

(1) $$\begin{array}{c} \%\text{Inhibition}=\left[\frac{{\text{Abs}}_{\text{control}}-{\text{Abs}}_{\text{extracts}}}{{\text{Abs}}_{\text{control}}}\right]\times 100. \end{array}$$

Concentrations of extracts resulting in 50% inhibition of enzyme activity (IC_50_) were determined graphically.

### 2.6. Mode of *α*-Amylase Inhibition

The mode of inhibition of *α*-amylase by the leaf extract was conducted using the extract with the lowest IC_50_ according to the modified method described by Ali et al. [18]. Briefly, 250 *μ*L of the extract (5 mg/mL) was preincubated with 250 *μ*L of *α*-amylase solution for 10 min at 25°C in one set of tubes. In another set of tubes *α*-amylase was preincubated with 250 *μ*L of phosphate buffer (pH 6.9). 250 *μ*L of starch solution at increasing concentrations (0.30–5.0 mg/mL) was added to both sets of reaction mixtures to start the reaction. The mixture was then incubated for 10 min at 25°C and then boiled for 5 min after the addition of 500 *μ*L of DNS to stop the reaction. The amount of reducing sugars released was determined spectrophotometrically using a maltose standard curve and converted to reaction velocities. A double reciprocal plot (1/*v* versus 1/(*S*)) where *v* is reaction velocity and (*S*) is substrate concentration was plotted. The type (mode) of inhibition of the crude extract on *α*-amylase activity was determined by analysis of the double reciprocal (Lineweaver-Burk) plot using Michaelis-Menten kinetics [19].

### 2.7. Alpha-Glucosidase Inhibitory Assay

The effect of the plant extracts on *α*-glucosidase activity was determined according to the method described by Kim et al. [20], using *α*-glucosidase from *Saccharomyces cerevisiae*. The substrate solution p-nitrophenyl glucopyranoside (pNPG) was prepared in 20 mM phosphate buffer, and pH 6.9. 100 *μ*L of *α*-glucosidase (1.0 U/mL) was preincubated with 50 *μ*L of the different concentrations of the extracts (acetone, ethanol, and water) for 10 min. Then 50 *μ*L of 3.0 mM (pNPG) as a substrate dissolved in 20 mM phosphate buffer (pH 6.9) was then added to start the reaction. The reaction mixture was incubated at 37°C for 20 min and stopped by adding 2 mL of 0.1 M Na_2_CO_3_. The *α*-glucosidase activity was determined by measuring the yellow-colored paranitrophenol released from pNPG at 405 nm. The results were expressed as percentage of the blank control.

Percentage inhibition is calculated as

(2) $$\begin{array}{c} \%\text{Inhibition}=\left[\frac{{\text{Abs}}_{\text{control}}-{\text{Abs}}_{\text{extract}}}{{\text{Abs}}_{\text{control}}}\right]\times 100. \end{array}$$

Concentrations of extracts resulting in 50% inhibition of enzyme activity (IC_50_) were determined graphically.

### 2.8. Mode of *α*-Glucosidase Inhibition

The mode of inhibition of *α*-glucosidase by the leaf extract was determined using the extract with the lowest IC_50_ according to the modified method described by Ali et al. [18]. Briefly, 50 *μ*L of the (5 mg/mL) extract was preincubated with 100 *μ*L of *α*-glucosidase solution for 10 min at 25°C in one set of tubes. In another set of tubes *α*-glucosidase was preincubated with 50 *μ*L of phosphate buffer (pH 6.9). 50 *μ*L of PNPG at increasing concentrations (0.63–2.0 mg/mL) was added to both sets of reaction mixtures to start the reaction. The mixture was then incubated for 10 min at 25°C, and 500 *μ*L of Na_2_CO_3_ was added to stop the reaction. The amount of reducing sugars released was determined spectrophotometrically using a paranitrophenol standard curve and converted to reaction velocities. A double reciprocal plot (1/*v* versus 1/[*S*]) where *v* is reaction velocity and [*S*] is substrate concentration was plotted. The type (mode) of inhibition of the crude extract on *α*-glucosidase activity was determined by analysis of the double reciprocal (Lineweaver-Burk) plot using Michaelis-Menten kinetics [19].

### 2.9. Statistical Analysis

Statistical analysis was performed using GraphPad Prism 5 statistical package (GraphPad Software, USA). The data were analysed by one way analysis of variance (ANOVA) followed by Bonferroni test. All the results were expressed as mean ± SE for triplicate determinations.

## 3. Results

Different extracts were obtained from *Morinda lucida* leaves from the different solvents (ethanol, acetone, and water) employed. Aqueous extract has the highest percentage yield of 9.99%, followed by acetone (7.30%) and ethanol (6.36%).

The phytochemical composition of the *Morinda lucida* extracts of acetone, ethanol, and water indicated the presence of flavonoids, tannins, and reducing sugar in all the extracts while saponins was detected only in the water extract (Table 1).

**Figure 1:**
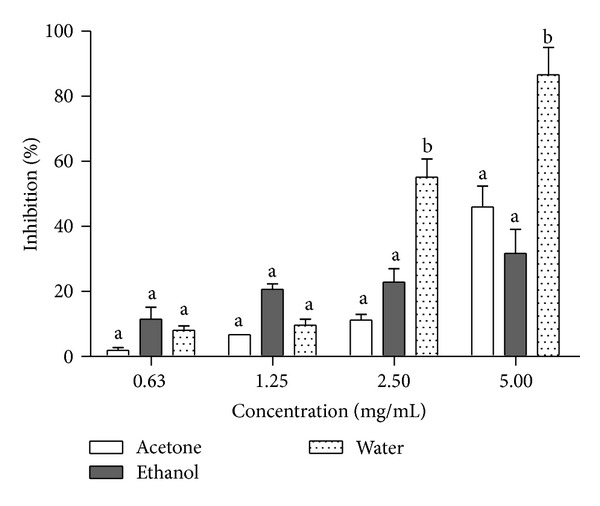
Inhibitory potency of *M. lucida *extract against *α*-amylase activity. The values are expressed as means ± SEM of triplicate tests. Means not sharing a common letter at the same concentration were significantly different (*P* < 0.05).

**Figure 2:**
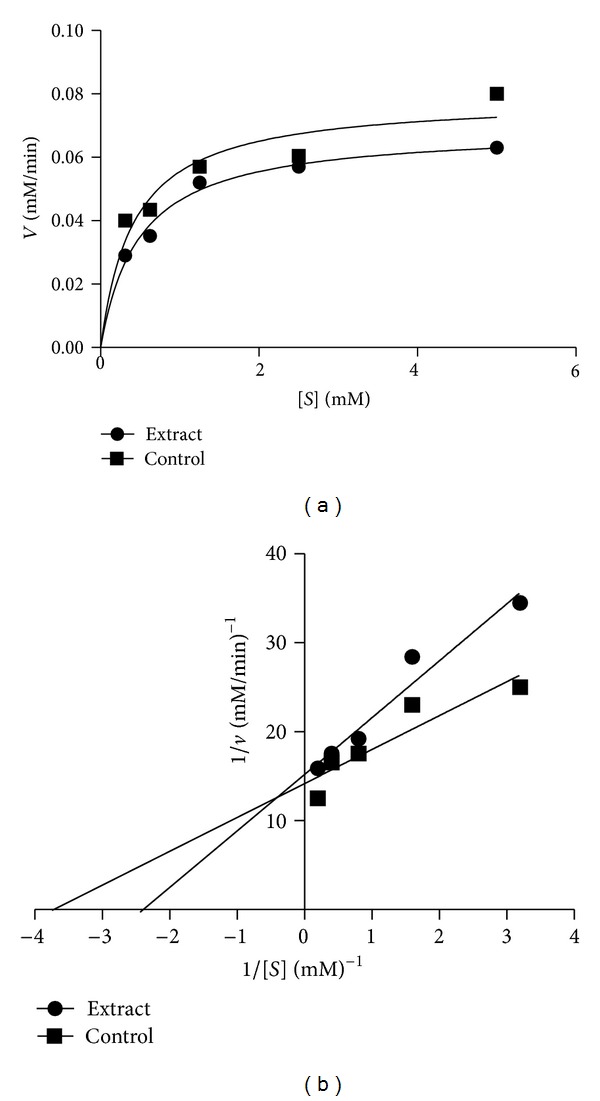
Mode of inhibition of *α*-amylase by aqueous extract of *M. lucida.* (a) Michaelis-Menten plot and (b) Lineweaver-Burk plot.

Alpha-amylase inhibition potential of the *Morinda lucida* extracts was determined (Figure 1). The inhibition of *α*-amylase by all the extracts at lower concentrations (0.63–1.25 mg/mL) showed no significant difference from one another but at higher concentrations (2.5–5 mg/mL), the inhibitory potential of aqueous extract was significantly different (*P* < 0.05) when compared to other extracts. Extrapolation of *α*-amylase effectiveness from the dose-response curve showed that aqueous extract contained the most potent *α*-amylase inhibitor with an IC_50_ value of 2.30 mg/mL (Table 2). The mode of inhibition of the aqueous extract of *Morinda lucida* leaf on *α*-amylase activity was determined using the Lineweaver-Burk plot which showed that the extract displayed a near competitive inhibition of the enzyme activity (Figure 2).

**Figure 3:**
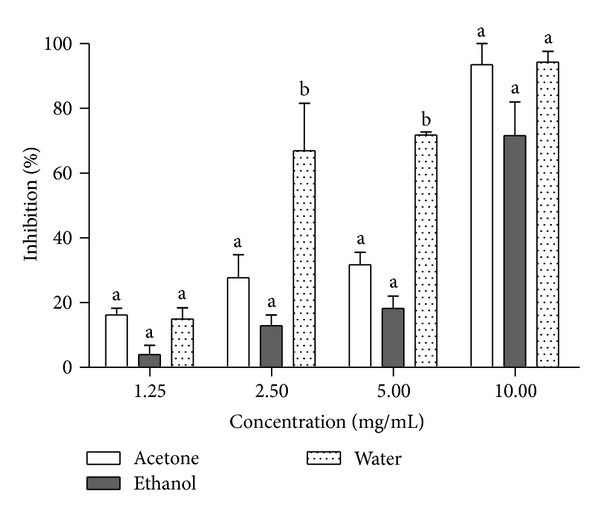
Inhibitory potency of *M. lucida *extract against *α*-glucosidase activity. The values are expressed as means ± SEM of triplicate tests. Means not sharing a common letter at the same concentration were significantly different (*P* < 0.05).

**Figure 4:**
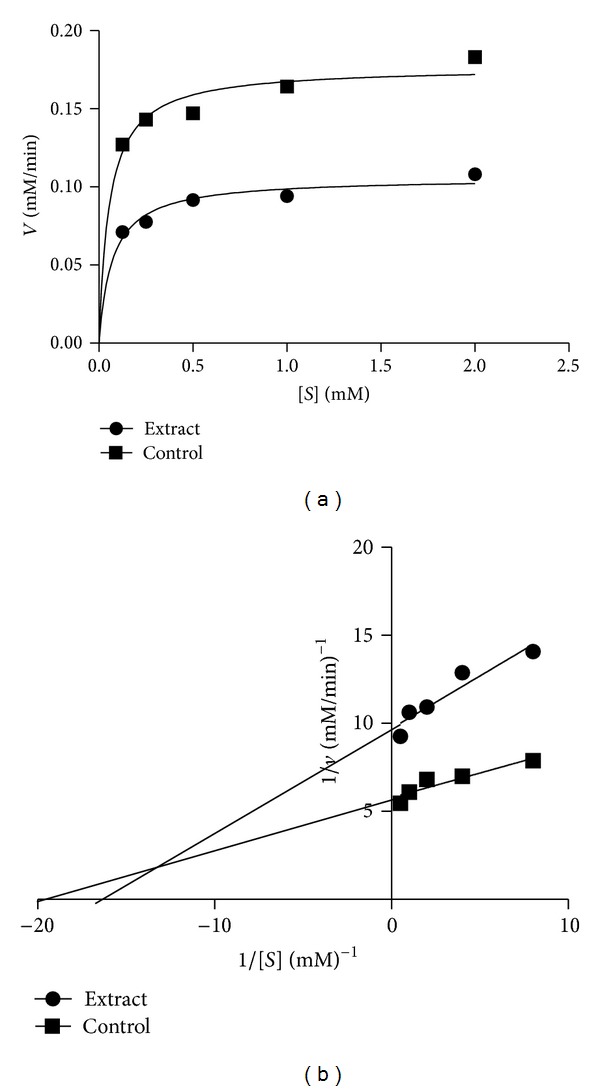
Mode of inhibition of *α*-glucosidase by aqueous extract of *M. lucida.* (a) Michaelis-Menten plot and (b) Lineweaver-Burk plot.

Alpha-glucosidase inhibitory potential of *Morinda lucida* leaf extracts was also determined (Figure 3). There was no significant difference among all the extracts at the lowest (1.25 mg/mL) and highest (10 mg/mL) concentrations. However, the aqueous extract exhibited the highest inhibitory potential on the enzyme which was significantly different (*P* < 0.05) when compared to ethanol and acetone extracts at the concentration of 2.50 mg/mL and 5 mg/mL. Also, investigation on *α*-glucosidase effectiveness showed a characteristic inhibition in which aqueous extract was also the most potent *α*-glucosidase inhibitor with an IC_50_ value of 2.00 mg/mL (Table 2). The mode of inhibition of the aqueous extract of *Morinda lucida* leaf on *α*-glucosidase was determined using the Lineweaver-Burk plot which displayed a mixed noncompetitive inhibition of the enzyme (Figure 4).

## 4. Discussion

Inhibitors of *α*-glucosidase delay the breaking down of carbohydrate in the small intestine and diminish the postprandial blood glucose excursion in a person suffering from diabetes [11]. One of the strategies and methods adopted to cure diabetes mellitus involves the inhibition of carbohydrate digesting enzymes such as *α*-amylase and *α*-glucosidase in the gastrointestinal glucose absorption thereby lowering postprandial glucose level [21]. This is an attempt to search for alternative drugs from medicinal plants with increased potency and lesser adverse effects than existing drugs [12–14].

In this study, the effect of *Morinda lucida* leaf extracts on the activities of *α*-amylase and *α*-glucosidase was evaluated. The plant extract showed potent inhibition of *α*-amylase activity. This result is in agreement with previous reports which indicated that excessive inhibition of pancreatic *α*-amylase could result in the abnormal bacterial fermentation of undigested carbohydrates in the colon and therefore mild *α*-amylase inhibition activity is desirable [22]. Lineweaver-Burk plot also showed that aqueous extract of this plant inhibit *α*-amylase competitively. This suggests that the active components in the extract compete with the substrate for binding to the active site of the enzyme thereby preventing the breaking down of oligosaccharides to disaccharides [13, 23].

As for *α*-glucosidase, the aqueous extract exhibits strong inhibition towards the activity of the enzyme. This is in line with reports of Kwon et al. [24] that natural *α*-glucosidase inhibitors from plants had been shown to have a strong inhibition activity against *α*-glucosidase and therefore can be potentially used as an effective therapy for postprandial hyperglycemia with minimal side effects. The mixed noncompetitive mode of inhibition obtained from the Lineweaver-Burk plot point to the fact that the active components in the extract do not compete with the substrate for binding to the active site rather the inhibitors bind to a separate site on the enzyme to retard the conversion of disaccharides to monosaccharides [14, 25].

Previous studies on the *in vivo* antidiabetic potential of *Morinda lucida* leaves in Wistar rats concluded that the extracts of this plant possessed strong glucose-lowering property in both alloxan and streptozotocin-induced diabetic rats [5, 6] but the mechanism of action remained elusive. The present study suggests that one of the mechanisms by which *Morinda lucida* exhibited its hypoglycemic potential is through the inhibition of pancreatic *α*-amylase and intestinal *α*-glucosidase of the animals used in previous studies.

This inhibitory activity of the *Morinda lucida* leaf extract might be due to the presence of several phytochemicals such as flavonoids, saponins, and tannins in it. Previous studies on *α*-amylase and *α*-glucosidase inhibitors isolated from medicinal plants suggest that several potential inhibitors belong to flavonoid class which has features of inhibiting *α*-amylase and *α*-glucosidase activities [11].

## 5. Conclusion

It can be concluded that, out of all the extracts of *Morinda lucida* tested for *α*-amylase and *α*-glucosidase inhibitory potential, aqueous extract displayed most the effective inhibition and this might be due to synergistic effect of the phytochemical constituents present in it. This study also suggests that one of the mechanisms by which this plant displayed its antidiabetic potential is by the inhibition of *α*-amylase and *α*-glucosidase. However, further study is needed to isolate the active principle(s) in this plant which is responsible for this activity.

## Figures and Tables

**Table 1:** Phytochemical composition of acetone, ethanol, and aqueous extracts of *Morinda lucida*.

Phytochemicals	Extracts		
	Acetone	Ethanol	Water
Anthraquinones	−	−	−
Flavonoids	+	+	+
Reducing sugar	+	+	−
Saponins	−	−	+
Steroids	+	+	−
Tannins	+	+	+
Terpenoids	−	+	+

(+): present; (−): not detected.

**Table 2:** IC_50_ values for *α*-amylase and *α*-glucosidase inhibitory potential of *M. lucida* leaf extracts.

Extracts	IC_50_ (mg/mL)	
	*α*-amylase	*α*-glucosidase
Acetone	5.85 ± 0.20^a^	6.60 ± 0.36^a^
Ethanol	9.10 ± 0.55^b^	8.15 ± 0.72^a^
Water	2.30 ± 0.08^c^	2.00 ± 0.05^b^

The values are expressed as means ± SEM of triplicate tests. Means down vertical column not sharing common letter are significantly different (*P* < 0.05).
